# Pediatric Therapeutic Plasma Exchange: Characterization of Practice, Epidemiology, and Safety Profile at a Children's Hospital in the United States

**DOI:** 10.1002/jca.70128

**Published:** 2026-05-01

**Authors:** Benjamin C. Lee, Brian D. Adkins, Ayda Javanbakht, Hung S. Luu, Daniel K. Noland

**Affiliations:** ^1^ Division of Hospital Medicine, Department of Pediatrics University of Texas Southwestern Medical Center Dallas Texas USA; ^2^ Children's Health Dallas Texas USA; ^3^ Department of Pathology University of Texas Southwestern Medical Center Dallas Texas USA

**Keywords:** apheresis complications, kidney disease, neurological diseases, pediatric, pediatric apheresis, plasma exchange, solid organ transplant rejection

## Abstract

There is a need to better understand the indications and safety profiles for therapeutic plasma exchange (TPE) in children. We aimed to assess pediatric TPE practice at a large academic center by retrospective chart review from 2011 to 2022. Patient demographics and clinical information including American Society for Apheresis (ASFA) category were analyzed. The cohort consisted of 438 patients, 52.1% female with a median age of 11.4 years, who underwent 3385 TPE procedures. The adverse reaction rate was 6.8%, with hypotension being most common. Tandem circuits were used in 3.9% of procedures, and the adverse reaction rate was significantly higher, 16.1% (*p* = < 0.05). Cryoprecipitate transfusion occurred in 29.0% of procedures for hypofibrinogenemia (median treatment number 3) and 19.1% of procedures required an RBC prime. Our findings highlight contemporary practical considerations for running a pediatric apheresis service, provide insight into managing tandem procedures, and may provide guidance for future research endeavors and clinical practice.

## 1 Introduction

Therapeutic plasma exchange (TPE) is an apheresis procedure designed to remove and modify plasma constituents. While TPE is used in both adult and pediatric patient populations, literature specifically characterizing pediatric practice remains limited. This gap is significant because pediatric patients present unique challenges including differences in size, disease presentation, and physiology, often necessitating tailored approaches to both the technical execution and clinical monitoring [1].

Pediatric patients may be at greater risk for procedure‐related complications due to volume shifts and low calcium from citrate toxicity, with some groups reporting higher rates of adverse reactions than adult patients (6%–55%) [1, 2, 3]. While there is much overlap, there are distinct differences between the two groups in terms of TPE technical performance and indications for treatment [4]. The American Society for Apheresis (ASFA) guidelines do not provide specific pediatric categorization, underscoring the relative paucity of data in this area [5]. Finally, given advancements in pediatric critical care, more patients are undergoing tandem procedures incorporating multiple circuits such as extracorporeal membrane oxygenation machines (ECMO) or continuous dialysis, which offer specific challenges related to fluid shifts, anticoagulation needs, and calcium supplementation [6].

There are limited prior studies assessing contemporary American TPE practice in pediatrics, and there is an ongoing need for insight into running an apheresis service at a pediatric hospital. As indications for TPE in pediatrics continue to expand, it is necessary to understand the current indications and practical aspects of a high‐volume inpatient apheresis service performing regular TPE. The aims of this study are fourfold: (1) categorize apheresis patients based on disease and current ASFA guidelines, (2) calculate a precise adverse reaction rate, (3) calibrate calcium dosing and fibrinogen/plasma replacement, and (4) establish a database for future questions developed from this initial analysis.

## 2 Materials and Methods

After receiving institutional board review approval from the University of Texas Southwestern Medical Center (STU‐2023‐0446) and site approval from Children's Health, apheresis billing information from January 1, 2011 to January 31, 2022, was reviewed to identify individuals who underwent TPE at Children's Health Dallas, a 496‐bed tertiary care children's hospital. Patients over 18 years of age at the time of the first procedure were excluded. Patient demographics, location of procedure, reason for TPE, number of exchanges, exchange volumes, replacement fluid type, equipment issues, laboratory studies, adverse events, interventions, concurrent ECMO or dialysis, and disposition were reviewed from the procedure note.

Procedures used the Spectra Optia Apheresis System (Terumo Blood and Cell Technologies, Lakewood, Colorado, USA). Our service requires central access with recommended double‐lumen dialysis‐compatible catheter sizing based on age: 0–2 years (7–8 French), 3–12 years (8–11 French), and 12 and older (11–14 French). Acid citrate dextrose solution was used as our anticoagulant for TPE procedures and calcium chloride (CaCl) was standardly provided for calcium replacement. From January 2011 through December 2021 calcium replacement was provided based on volume exchanged as follows: 500–750 mg CaCl for TPE < 1000 mL, 1000 mg for TPE 1250–2999 mL, and 1500 mg CaCl for TPE > 3000 mL. In December 2021 calcium supplementation was changed to 250 mg CaCl per full liter exchanged. CaCl is administered by injecting the medication using a syringe into albumin bottles or infused over the course of the procedure with a medication pump if blood products are used as exchange fluids. Replacement fluid options were 5% albumin, plasma or cryoprecipitated antihemophilic factor (CRYO) depending on the patient's indication for TPE, lab results, and current health baseline. RBC prime was performed for patients whose extracorporeal volume (ECV) was greater than 15% of their total blood volume or if the pre‐procedure hematocrit was less than 21%. The fibrinogen trigger for transfusion was 120 mg/mL for the near entirety of the study period and changed to 100 mg/mL in fall of 2021.

The indication for each patient's initial TPE procedure was categorized using the most recent ASFA guidelines [5]. Diagnoses were also evaluated separately for ASFA category and then grouped based on common indications for evaluation of prevalence ex. all solid organ transplantation desensitization and rejection combined as one grouping titled solid organ transplant‐related disorders or antibody negative and anti‐N‐methyl‐D‐aspartate (NMDA) receptor encephalitis grouped as autoimmune encephalitis. Pre‐TPE fibrinogen, ionized calcium, hemoglobin, and hematocrit values were collected from laboratory results performed within 24 h prior to each procedure. High or low clinical laboratory values were determined using the institutional reference range per age.

During the time investigated, our institutional apheresis service was managed by three attending pathologists (BDA, DKN, HSL). Our practice is as follows: a physician is on call 24/7 and apheresis procedures are performed 7 days a week with timing based on acuity, with high acuity patients treated overnight and lower acuity patients treated starting the next day. Apheresis staff (approximately 10 nurses) are contracted through our regional blood supplier, Carter Bloodcare (Bedford, Texas). The apheresis service is consulted to determine if apheresis is indicated, to provide guidance on access, and to help determine treatment course. The pathologist is in charge of ordering TPE medications, determining volume of exchange, anticoagulation and replacement fluid selection, and procedural oversight, while the remainder of patient care, such as daily laboratory assessment, is performed by the primary team. We required pre‐procedure hemoglobin/hematocrit, fibrinogen, and ionized calcium testing. Other treating primary and subspecialty services were characterized. Each treating service was noted for patients managed by multiple services, example a nephrology patient receiving treatment in the pediatric intensive care unit.

Data was stored in an Excel spreadsheet (Microsoft, Redmond, WA, USA) on a secure, password protected network. The patient cohort was divided equally among four authors (BDA, BCL, HSL, and DKN), and the electronic medical records (Epic Systems Corporation, Verona, Wisconsin, USA) were reviewed with 10% of patients audited for accuracy (BDA). Descriptive statistics were performed using GraphPad Prism (GraphPad Prism 10.6.1, GraphPad Software LLC, Boston, MA, USA). Normality was assessed using the Kolmogorov–Smirnov test and results are presented as the median with the interquartile range (IQR), unless otherwise specified. Groups were compared using Fisher's exact test and a *p* value < 0.05 was considered statistically significant.

## 3 Results

### 3.1 Demographics

A total of 438 patients underwent 3385 procedures. The female to male ratio was 1.1. The median age was 11.4 years (IQR 5–15.2), and the median weight was 40.0 kg (IQR 19.1–60.0). The median number of courses was 1 (IQR 1–1, range 1–12) with a median number of total procedures of 5 (IQR 5–7, range 1–110) (Table 1).

**TABLE 1:** Demographic characteristics of an 11‐year cohort of pediatric patients undergoing therapeutic plasma exchange.

Characteristics	Description
Total patients	438
Male	47.9% (210/438)
Female	52.1% (228/438)
Patient age	Median = 11.4 years (IQR 5–15.2, range 6 days—19.0 years)
< 2	11.4% (50/438)
2–10	31.1% (136/438)
> 10	57.5% (252/438)
Patient weight (kg)	
< 10	5.0% (22/438)
10–20	21.2% (93/438)
> 20	73.7% (323/438)
Procedures	3385
Medium number of courses per patient	1 (IQR 1–1, range 1–12)
Median number of procedures per course	5 (IQR 5–7, range 1–110)
ASFA 2023 category	
Category I	39.5% (173/438)
Category II	42.2% (185/438)
Category III	17.6% (77/438)
Category IV	0.0% (0/438)
Not categorized	0.7% (3/438)

**FIGURE 1:**
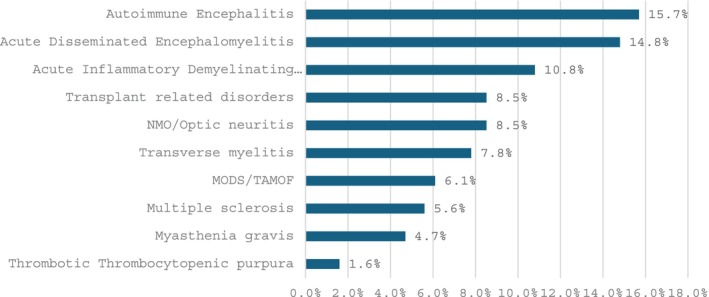
Indication for therapeutic plasma exchange for pediatric TPE patients by indication. Other indications not listed (15.9%). Abbreviations are as follows NMO, neuromyelitis optica; MODS/TAMOF multi‐system organ dysfuntion/Thrombocytopenia‐associated multiple‐organ failure.

Patient procedures were categorized by the current ASFA 2023 guidelines for TPE: category I, 39.5% (173/438); category II, 42.2% (185/438); category III, 17.6% (77/438); category IV 0% (0/438); not categorized 0.7% (3/438) [5]. Most patients were treated for autoimmune neurologic conditions: autoimmune encephalitis (15.7%, 70/438), acute disseminated encephalomyelitis (14.8%, 66/438), and acute inflammatory demyelinating polyneuropathy (10.8%, 48/438), followed by solid organ transplant‐related disorders (8.5% 38/438) (Figure 1).

Treating services per procedure were as follows: neurology, 61.7% (2090/3385), hospitalist medicine, 53.9% (1823/3385), nephrology, 20.1% (681/3385), pediatric intensive care unit, 19.5% (660/3385), hematology/oncology, 6.1% (208/3385), gastroenterology, 4.8% (162/3385), cardiovascular intensive care unit, 1.7% (59/3385), psychiatry, 1.2% (41/3385), rheumatology, 0.9% (32/3385), endocrinology, 0.6% (19/3385), infectious disease, 0.4% (14/3385), pulmonary, 0.4% (13/3385), ophthalmology, 0.3% (10/3385), and neonatal intensive care unit, 0.1% (5/3385).

### 3.2 Technical Considerations

Most procedures consisted of a 1.1 plasma volume exchange (median 1.1, IQR 1.1–1.1, range 0.3–1.6). All patients had central line access with a dialysis‐compatible central venous catheter (CVC), or the ECMO (Extracorporeal Membrane Oxygenation) circuit was utilized. CVC line issues, including poor flow or loss of access, occurred in 10.7% (47/438) of patients and impacted 2.4% (81/3385) of procedures. Access issues tended to occur in smaller, younger patients (median weight 31.5 kg, IQR 13.7–56; median age 7.7 years, IQR 3.4–14.4) and earlier procedures (mode procedure 1, median procedure 3). No fistula use was documented.

**FIGURE 2:**
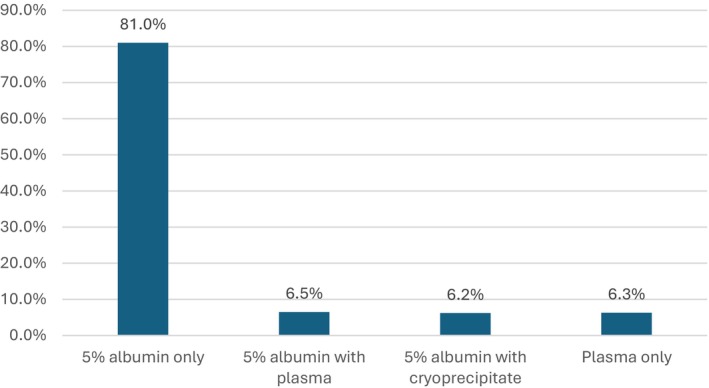
Exchange fluid for TPE in pediatric patients.

The majority of procedures used 5% albumin only as an exchange fluid, though many patients received fresh frozen plasma (FFP) or albumin and FFP (Figure 2). CRYO was used as a replacement fluid for 29.0% (127/438) of patients and in 6.3% (209/3385) of procedures for hypofibrinogenemia. CRYO transfusion tended to be required in the first few procedures (mode procedure 2, median procedure 3). An RBC prime was required in 19.1% (647/3385) of procedures. The need for CRYO transfusion did not show a weight or age association (median weight 37.9 kg, IQR 19.1–56.5; median age 10.72 years, IQR 4.8–14.9).

### 3.3 Laboratory Assessment

Clinical laboratory values were assessed prior to TPE. Amongst the cohort, 61.4% (269/438) had at least one baseline laboratory abnormality: anemia (98.5%, 265/269), hypofibrinogenemia (14.9%, 40/269), or abnormal ionized calcium testing (27.1%, 73/269). The remaining patients were evaluated for the development of lab abnormalities. A majority of patients, 76.9% (130/169), developed at least one laboratory abnormality during their treatment course as follows: anemia 76.9% (100/130), hypofibrinogenemia 66.2% (86/130), and ionized calcium abnormalities 19.2% (25/130).

**FIGURE 3:**
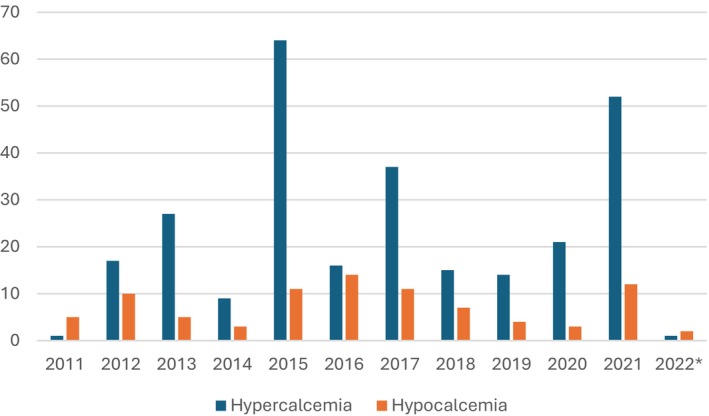
Incidence of pre‐procedure calcium abnormalities over the time period investigated. *The time period investigated in 2022 was limited to 31 days.

The incidence of pre‐procedure ionized calcium abnormalities was charted year over year, with hypercalcemia occurring more frequently than hypocalcemia (Figure 3). In December 2021, our calcium dosing was lowered to reduce pre‐procedural hypercalcemia and the incidence of pre‐procedure abnormal calcium levels was low in the 2 months investigated, with 7 hypercalcemia cases and 2 hypocalcemia cases.

### 3.4 Adverse Reactions

**FIGURE 4:**
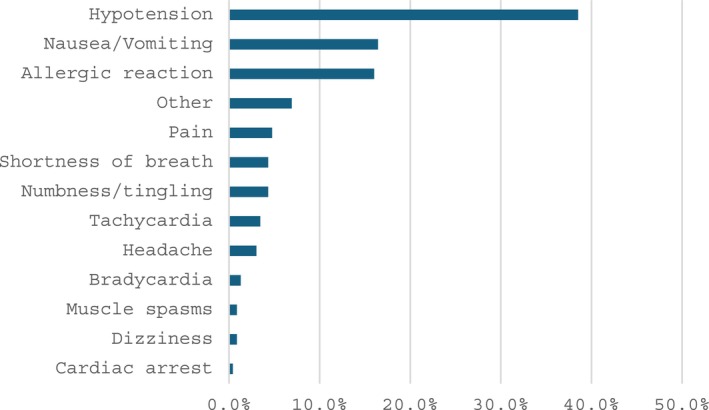
Percentage of adverse reactions in TPE patients with procedure‐related reactions. Other category symptoms include non‐specific findings such as agitation, pain, flushing, and discomfort.

Clinical adverse reactions occurred in 6.8% (231/3385) of procedures. Amongst those with reactions, 42.9% (99/231) were receiving a blood product as part of TPE, with some patients receiving multiple products: 64.6% plasma (64/99), 34.4% (34/99) RBC, and 10.1% (10/99) CRYO. The most common adverse reaction was hypotension (Figure 4) and no serious bleeding events were documented. Amongst patients with allergic symptoms (urticaria and pruritis), most received blood products with the procedure (70.2%, 26/37).

Intraprocedural calcium abnormalities were measured in 0.6% (19/3385) of treatments with most being hypocalcemia only (13/19), followed by hypercalcemia only (5/19), or both (1/20). No intraprocedural calcium abnormalities were measured after procedural dosing changed. Amongst the 10 patients with intraprocedural hypocalcemia (3 patients had multiple occurrences) the median weight was 55.3 kg (IQR 31.5–66.3), median age was 15.3 years (IQR 8.6–17.1) and 80.0% (8/10) were undergoing TPE using blood products. Liver failure as defined by indication was present in 50.0% (5/10) of these patients.

Two severe adverse events were reported: (1) asystole secondary to hypocalcemia in a 4‐week‐old male on ECMO undergoing TPE for acute liver failure with an RBC prime and appropriate calcium chloride supplementation, who was resuscitated but died 2 days later from liver failure and (2) a 9‐year‐old female with immune thrombotic thrombocytopenic purpura (iTTP) developed a severe allergic reaction requiring epinephrine and oxygen via a non‐rebreather mask.

### 3.5 Tandem Procedures

Tandem procedures were performed in a minority of cases, 3.7% (124/3385), and were associated with a significantly higher incidence of adverse reactions, 16.1% (20/124) versus patients undergoing TPE alone, 6.5% (211/3261) (*p* = < 0.05). Procedures were performed in tandem with ECMO, 61.3% (76/124); continuous dialysis, 60.5% (75/124); or both, 21.7% (27/124). No tandem hemodialysis was reported. Adverse reactions included hypotension (7/20), allergic reaction (3/20), emesis (1/20), oxygen desaturation (1/20), hypertension (1/8), urticaria (1/8), and asystole (1/8). Intraprocedural calcium abnormalities were measured in 40% (8/20) of these patients, with most being hypocalcemia only (6/8), followed by hypercalcemia only (1/8), or both (1/8). No bleeding events were reported.

## 4 Discussion

Findings from our large cohort demonstrate that TPE is well‐tolerated and a collaborative approach to patients can yield a low rate of adverse events. We also demonstrated strict adherence to ASFA recommendations based on the current ASFA guidelines, with 82% of our patients having Category I and II indications, which is higher than previously reported literature whose ranges were from 35% to 71% [7, 8, 9]. Having patients with uncategorized indications is common, but our study had 0.7% as compared to 5%–13% found in other studies [7, 8, 9]. Our data suggest increased utilization of TPE in pediatric patients, in contrast to earlier work suggesting low levels of TPE use, particularly in Category I indications [10].

Our most frequent indications for TPE were the same as a recent adult investigation: autoimmune neurologic conditions and transplantation related indications [5, 11]. Our most common indication amongst autoimmune neurologic disorders was autoimmune encephalitis, which contrasts with adult data where neuromyelitis optica was most common [11]. Although this is similarly common in our cohort, autoimmune encephalitis tends to occur in younger individuals, median age 23.7 years (IQR = 14.2–31.0 years), which may lead to a relative enrichment of cases amongst our patients [12]. Although many cases had an anti‐NMDAR antibody identified, given the severity of these presentations, our group tends to initiate TPE while awaiting antibody testing. Many prior pediatric studies showed more utilization of TPE for renal conditions and this may reflect institutional culture or regional incidence of disease [7, 13, 14]. Many of our patients were being managed by nephrology, as we are often consulted by that service to manage transplant related conditions. Given the increase in biologic medications for managing transplant patients such as monoclonal antibody therapies, it is important to coordinate and time treatments in order to optimize desensitization or rejection protocols.

Central line access was effective and safe in our cohort. Line placement is generally well‐tolerated, but it should be noted that it is an invasive procedure which can result in morbidity and mortality; patients may develop line associated infections with prolonged use [15]. Equipment and central line issues affected only 2.4% of our procedures, which is similar to other studies, though we only had mild flow rate‐related problems rather than more serious adverse reactions [8, 16]. Access issues were seen more often in younger patients during their first session of TPE. Typically, young patients have smaller diameter catheters, which may increase the risk of occlusion. Minimum catheter size per age was discussed between transfusion medicine and surgery services and a standard practice was put in place, which in our experience has improved performance of TPE. Developing a process with institutional colleagues helps ensure appropriate lines are placed and allows for more streamlined initiation of TPE, as this is frequently a barrier to initiating treatment. Given the risk of access failure, our group does not perform TPE procedures peripherally and recommends central line placement to ensure reliable TPE access.

Few studies discuss specific abnormal pre‐procedure laboratory results and frequently lump them with equipment issues and clinical reactions as overall procedural complications. Several studies had overall complication rates of 5%–19%, of which 5%–20% were hypocalcemia [16, 17, 18]. In our study, the most common pre‐TPE abnormal lab findings were anemia or hypofibrinogenemia. This may be due to the combination of underlying disease, blood draws for laboratory testing, and apheresis procedures. Further, although calcium levels may change during the procedure, physiologic changes in electrolytes can be accommodated for more rapidly than reconstitution of clotting factors or erythropoiesis in response to iatrogenic blood loss.

Intraprocedural hypocalcemia due to citrate toxicity was found in a minority of our patients; however, intraprocedural monitoring of labs was rare outside of intensive care unit patients or those with tandem circuits. Given the challenges in monitoring ionized calcium levels in children and the difficulties in recognizing and communicating symptoms of hypocalcemia, all patients received intravenous calcium chloride during apheresis as a preventative measure. We saw more hypercalcemia in their post‐apheresis labs. Although some of our patients were asymptomatic, we implemented a standardized approach with lower initial doses to minimize the incidence of pre‐procedural hypercalcemia in our patients. We also continued to fine‐tune our calcium dosing and citrate anticoagulation ratios in patients based on exchange fluid and baseline liver function. Calcium labs have been followed in the intervening years and no severe hypocalcemia reactions have been encountered (data not shown as outside of the study period, internal quality data). A nuanced approach is often necessary to prevent adverse effects of hypocalcemia, and treating teams should be cognizant of the issues surrounding calcium dosing for TPE patients.

With no documented instances of major bleeding in 3385 plasma exchanges over 11 years, we have decreased our fibrinogen threshold for transfusion when managing stable patients without increased risk of bleeding (example diffuse alveolar hemorrhage). Our transfusion medicine colleagues at the adult hospitals on campus use a fibrinogen cut‐off of 80 mg/mL unless patients are on systemic anticoagulation and have recently characterized the safety profile of this practice [19]. At this time, the 100 mg/mL cut‐off remains our conservative approach, and we will also use plasma as an exchange fluid in patients in the post‐operative period.

Allergic reactions were common in our cohort. The most common clinical apheresis adverse reactions in other studies were hypotension, nausea and vomiting, and urticaria/pruritis, which was also demonstrated in our cohort [7, 14, 16, 17, 18]. When allergic reactions occurred, most of the time, blood products were used concurrently, thus suggesting a reaction to the blood product rather than an adverse event related to TPE, although ethylene oxide sensitivity to the apheresis kit cannot be ruled out. Another study at a large pediatric center reported rare adverse effects such as seizures, pneumothorax, stroke, and cardiac arrest, and while we only had 2 patients develop severe reactions during the procedure, this is noteworthy and may suggest that clinically tenuous patients begin apheresis in a critical care setting prior to transfer to a general or subspecialty floors [8].

Although a small percentage (3.7%) of our TPE procedures occurred in tandem, this subgroup had a significantly higher percentage of clinical adverse reactions compared to those who underwent TPE alone (16.1% vs. 6.5%, respectively). Hypotension and hypocalcemia were the most common adverse reactions observed in our patients who underwent TPE in tandem with dialysis and/or ECMO, which highlight issues related to volume shifts and citrate burden [20]. As many of these patients require citrate‐containing blood products or have reduced citrate metabolism due to liver failure, close monitoring, including intraprocedural calcium assessment, is warranted. Performance of tandem procedures can be successful only when treating teams work together and there is a clear understanding of circuit volumes, patient organ function, and timing of TPE. With multi‐disciplinary discussion and coordination, these critically ill patients may successfully undergo TPE.

Our study has several limitations. Patients were retrospectively identified from billing data, potentially excluding those without billing records. The study was conducted at a single tertiary‐care pediatric hospital in the United States, so findings may not be generalizable to other settings. TPE‐related complications were identified through procedure notes and may have been underreported if not documented. Additionally, the number of patients included in the study who received reduced CaCl dosing is low, which limits our ability to attribute improved calcium levels long‐term. Further, some changes may reflect individual practice, which can influence procedural characteristics based on the attending on service.

Future studies involving multiple sites could continue evaluating the safety profile and abnormal laboratory results, as well as investigate therapeutic outcomes of TPE for specific ASFA indications in pediatric patients. Procedural complications should be subdivided into equipment/line issues, laboratory abnormalities, and clinical reactions to study each factor. Adjustments in ASFA indication categories or diseases where TPE could be beneficial could necessitate recurrent review of TPE practice. TPE impact on long‐term clinical outcomes or adverse reactions is unknown and could be elucidated with longitudinal studies. National or international registries or databases could help address these areas of investigation.

## 5 Conclusion

TPE is a safe procedure and can be employed on a large scale in a pediatric center. Performing TPE concurrently with dialysis and/or ECMO was associated with a significantly increased rate of adverse reactions and close monitoring is necessary for optimal management. Our findings show that age and clinical condition are not exclusionary criteria for performing TPE, but treating teams should be aware of common issues which require a patient‐tailored approach.

## Funding

The authors have nothing to report.

## Disclosure

The authors have nothing to report.

## Conflicts of Interest

The authors declare no conflicts of interest.

## Data Availability

The data that support the findings of this study are available from the corresponding author upon reasonable request.
